# In-Hospital Mortality in Patients With Acute ST-Elevation Myocardial Infarction With or Without Mitral Regurgitation

**DOI:** 10.7759/cureus.23762

**Published:** 2022-04-02

**Authors:** Rafi Ullah, Farhat Shireen, Ahmad Shiraz, Sher Bahadur

**Affiliations:** 1 Cardiology, Lady Reading Hospital Peshawar, Peshawar, PAK; 2 General Surgery, Hayatabad Medical Complex Peshawar, Peshawar, PAK; 3 Epidemiology and Public Health, Khyber Institute of Child Health, Peshawar, PAK

**Keywords:** comorbidities, risk factors, in-hospital mortality, mitral regurgitation, st-elevation myocardial infarction

## Abstract

Background

Mitral regurgitation (MR) is a common complication in hospitalized cardiac patients with ST-segment elevation myocardial infarction (STEMI); however, the patient outcomes depend on various factors that vary across facilities and regions. There is an acute need to stratify STEMI patients by risk of in-hospital mortality. We conducted this study to compare the mortality of patients with acute STEMI with or without MR admitted to different units of the Cardiology Department at Lady Reading Hospital (LRH) in Peshawar.

Methods

In this prospective study, we compared the mortality rates of STEMI patients with and without MR from June 5 to October 30, 2021. All patients with different types of STEMI treated at LRH were enrolled in the study regardless of age and gender. ST-elevation was confirmed via electrocardiogram, and MR was confirmed via echocardiography. We excluded any patients with primary organic valve disease or congenital heart disease. We also collected patient demographic and clinical characteristics. We used IBM SPSS Statistics for Windows, Version 24.0 (IBM Corp., Armonk, NY) for statistical analyses.

Results

Our study population included 228 patients with a mean age of 62.4 ± 12.3 years. Most of the patients were men (n=140; 61.4%), and only 78 (38.6%) were women. The prevalence of MR was 29.4%. Hypertension was the most common comorbidity (63.6%), and inferior wall myocardial infarction (MI) was the most common type of MI (49.1%). Hypertension, prehospital cardiopulmonary resuscitation (CPR), and Killip class ≥ 2 were significantly associated with MR (p<.001). In-hospital mortality was 29.8%, significantly associated with MR (p=.0001). Patients who needed CPR prior to hospitalization and those with Killip class ≥ 2 were less likely to survive (p=.0001).

Conclusions

MR is common following MI, especially in cases of inferior wall MI. Patients with MR have a poorer prognosis than those without MR following MI, more so when combined with other comorbidities. Regarding its relation to MI complications, an assessment of the MR is necessary to make an appropriate decision for treatment.

## Introduction

In-hospital mortality is defined as deaths that occur during hospitalization, and it varies from disease to disease and the quality of health care services [1]. Despite advances in treatment, patients with acute myocardial infarction (MI) have a poor long-term prognosis. Hospitalized patients with ST-segment elevation MI (STEMI) have an increased mortality rate [2,3], and mitral regurgitation (MR) is a common complication of STEMI [4,5].

Acute STEMI is a life-threatening condition associated with cardiac diseases [6]. STEMI patients have increased 30-day mortality rates and high readmission rates [7]. Patients with STEMI usually present with MR, especially those undergoing angioplasty [5].

MR is associated with in-hospital mortality; however, multiple factors affect patient outcomes and vary by location. Globally, nearly half of the burden of ischemic heart disease occurs in Asia [8]. Mortality due to cardiac disease has significantly decreased in high-income countries, where timely availability of specialists and equipment in the hospital has positively impacted mortality rates [9]. Unfortunately, the same trend is not present in low-income countries - the incidence of in-hospital mortality is increasing in an environment that lacks adequate acute cardiac care facilities [10]. There is an urgent need to stratify STEMI patients by risk of mortality in such a resource-limited setting. Therefore, we conducted this study to compare the in-hospital mortality rates among STEMI patients with and without MR.

## Materials and methods

Study design and data collection

We conducted a cross-sectional study of patients with acute STEMI who presented to a Lady Reading Hospital, a public sector tertiary care hospital in Peshawar, from June 5 to October 30, 2021. All patients with STEMI were included in the study, regardless of the type of STEMI. STEMI was confirmed via electrocardiogram (ECG), where the elevation of >1 mm was considered positive. MR was confirmed on echocardiographs evaluated by qualified department cardiologists. Patients were excluded if they had known Ischemic MR prior to the index event or if they were found to have primary MR prior to MI diagnosis. Data were recorded in a structured proforma that collected demographic and clinical information. Demographic variables comprised age and gender, while clinical variables included the history of hypertension, pre-hospital CPR, and the presence of MR along with different types of STEMI.

ECG and echocardiography results were categorized as either STEMI with MR or STEMI without MR. Patients were followed until discharge from the hospital or in-hospital death to assess the rate of mortality. Our study protocol received technical and ethical approval from the College of Physicians and Surgeons in Pakistan. All participants and family members of deceased patients provided written informed consent to participate in the study.

Statistical analysis

We used IBM SPSS Statistics for Windows, Version 24.0. (IBM Corp., Armonk, NY) to analyze the study data. Descriptive statistics were determined for all variables in terms of mean, standard deviation, frequency, and percentage. We used the chi-square test to compare the impact of risk factors associated with survivorship and in-hospital mortality, and we considered p-values ≤ 0.05 as statistically significant.

## Results

**Figure 1:**
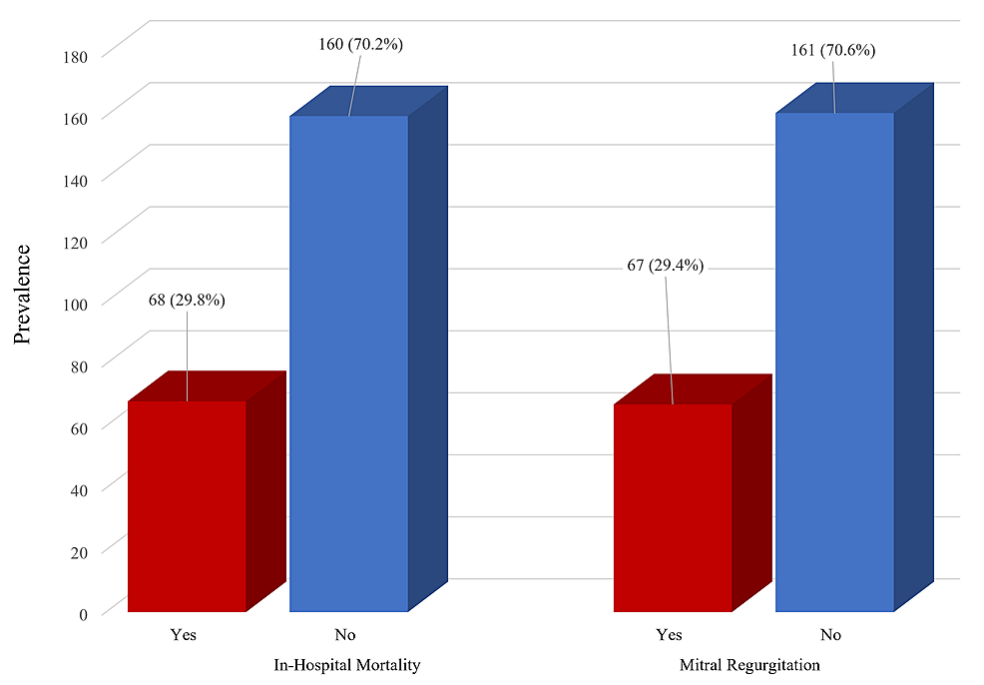
Prevalence of mitral regurgitation and in-hospital mortality MR, mitral regurgitation

Our study population consisted of 228 patients admitted with STEMI (mean age, 62.4 ± 12.3 years). Most patients were men (n=140; 61.4%), and 78 (38.6%) were women. More than half (63.6%) had a history of hypertension (Table 1). Twenty-three patients (10%) received prehospital cardiopulmonary resuscitation (CPR), and 44 patients (19.3%) were categorized as Killip class ≥ 2. One hundred twelve patients had inferior wall MI (the most common at 49.1%), followed by anterior wall MI (n=72; 31.6%) and posterolateral wall MI (n=44; 19.3%). The overall prevalence of MR was 67 patients (29.4%), while the in-hospital mortality was 68 (29.8%; Figure 1).

**Table 1:** Frequency of MR risk factors in patients with STEMI MR, mitral regurgitation; HTN, hypertension; CPR, cardiopulmonary resuscitation; MI, myocardial infarction; STEMI, ST-segment elevation myocardial infarction.

Clinical Characteristics		N	Percentage (%)
History of HTN	Yes	145	63.6
	No	83	36.4
Prehospital CPR	Yes	23	10.1
	No	205	89.9
Killip class ≥2	Yes	44	19.3
	No	184	80.7
Anterior wall MI	Yes	72	31.6
	No	156	68.4
Inferior wall MI	Yes	112	49.1
	No	116	50.9
Posterolateral wall MI	Yes	44	19.3
	No	184	80.7

In-hospital mortality was 55.9% in males and 44.1% in females (p=.26); moreover the patients with hypertension had higher proportion of mortality (64.7%) as compared to those without hypertension (35.3%) but the difference was not significant (p=.82). Patients who underwent prehospital CPR were significantly less likely to survive-only five such patients (3.1%) did so (p=.0001). the patients with in-hospital mortality (n=68), 44.1% patients were having Killip class ≥ 2 as compared to patients without heart failure or Killip class < 2 (p=.0001).

**Figure 2:**
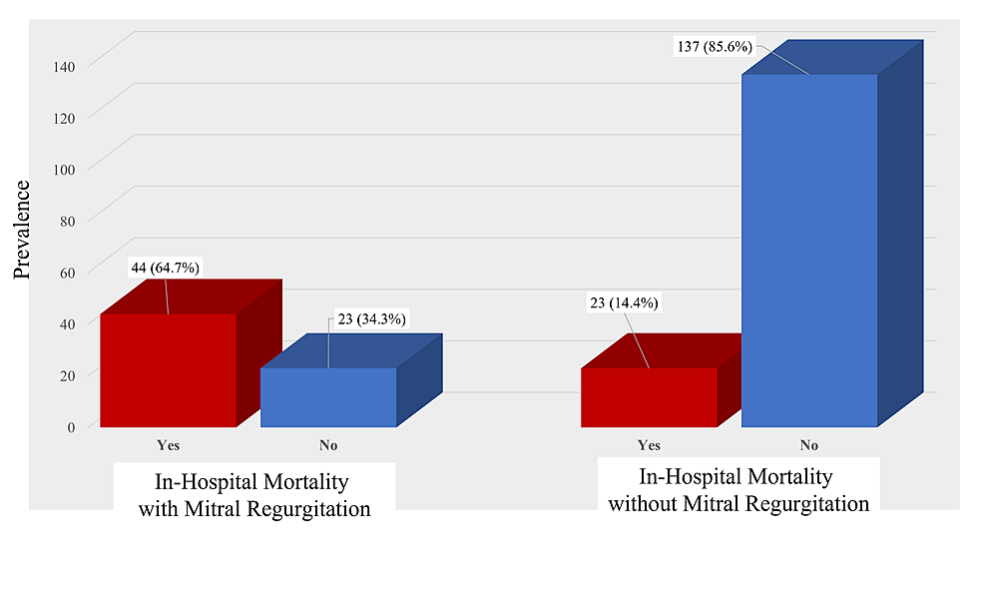
Comparison of in-hospital mortality among patients with MR and without MR (p=.0001) MR, mitral regurgitation

Fifty-one patients (31.9%) had anterior wall MI and survived to be discharged, while 21 patients (30.9%) had anterior wall MI and died in the hospital. Seventy-six patients (47.5%) had inferior wall MI and survived to be discharged, and 36 patients (52.9%) had inferior wall MI and died in the hospital (p=.45; Table 2). Posterolateral wall MI was less associated with in-hospital mortality than other MI variants. MR was significantly associated with in-hospital mortality: 44 patients with MR died in the hospital (64.7%) compared to the 23 with MR who survived (14.4%; p=.0001; Figure 2).

**Table 2:** Risk factors association with mitral regurgitation and its impact on in-hospital mortality MR, mitral regurgitation; HTN, hypertension; CPR, cardiopulmonary resuscitation; MI, myocardial infarction; STEMI, ST-segment elevation myocardial infarction

Associated risk factors of MR		In-Hospital Mortality		P-value
		Yes	No	
		n (%)	n (%)	
Gender	Male	38 (55.9%)	102 (63.8%)	0.26
	Female	30 (44.1%)	58 (36.2%)	
History of HTN	Yes	44 (64.7%)	101 (63.1%)	0.82
	No	24 (35.3%)	59 (36.9%)	
Prehospital CPR	Yes	18 (26.5%)	5 (3.1%)	0.0001
	No	50 (73.5%)	155 (96.9%)	
Killip class ≥2	Yes	30 (44.1%)	14 (8.8%)	0.0001
	No	38 (55.9%)	146 (91.2%)	
Anterior wall MI	Yes	21 (30.9%)	51 (31.9%)	0.88
	No	47 (69.1%)	109 (68.1%)	
Inferior wall MI	Yes	36 (52.9%)	76 (47.5%)	0.45
	No	32 (47.1%)	84 (52.5%)	
Posterolateral wall MI	Yes	8 (11.8%)	36 (22.5%)	0.06
	No	60 (88.2% )	124 (77.5%)	
Mitral Regurgitation	Yes	44 (64.7%)	23 (14.4%)	0.0001
	No	23 (34.3%)	137 (85.6%)	

## Discussion

Ischemic heart disease remains a leading cause of mortality and morbidity globally, despite advancements in the treatment of acute MI, including STEMI. The improved methods in the treatment of STEMI have significantly reduced the occurrence of cardiac shock. However, MR is a drastic adverse event commonly observed in cardiac patients, with a prevalence of over 30% of cases in Pakistan [11]. Our study aimed to determine the role of MR and other risk factors contributing to in-hospital mortality in STEMI patients.

The mortality rate of STEMI depends on treatments and patient risk factors. Proper interventions in these patients can reduce the mortality rate, but it increases to 50% without such interventions [12]. The mortality rate due to STEMI in our study was 29.8%, aligning with the 27% mortality rating in STEMI patients reported by Johnson et al. [12].

Risk factors for STEMI mortality include hypertension, diabetes, obesity, high cholesterol levels, and MR [11]. Hypertension was the most common risk factor for in-hospital mortality in STEMI patients, with a 63.6% prevalence in our study. Mentias et al. reported a similar proportion; 74.6% of STEMI patients in their study also had hypertension [4]. Similarly, our finding of inferior wall MI as the most prominent type of MI aligned with results from a study in Spain that reported inferior wall MI was most common in their population, significantly more common in men (27.9%) than women (24.9%; p>.05) [13].

The overall rate of prehospital CPR use among patients with STEMI was 10.1%, and the survival to discharge rate among patients who received CPR was 3.1% in our study. This percentage was lower than that reported by Moosajee et al. (7.5%) [14]. Another study of mortality in cardiac patients reported that 19.1% of patients who received CPR died [15]. The variability in survival among patients who required CPR could be due to the quality of care and delays in access to health services. The hospital where our study was conducted is one of the largest teaching hospitals in the province, providing health care to patients referred from peripheral facilities. When patients reached our hospital, their conditions had already worsened, which might explain the low success rate of CPR.

Patients with Killip class ≥ 2 had a positive correlation with in-hospital mortality. A long-term follow-up study revealed that mortality varies according to Killip class, where class 1 had 17.7%, class 2 had 27.3%, class 3 had 30.4%, and class 4 had 48.5% mortality [16]. This trend indicates that these patients commonly develop pulmonary complications, especially pulmonary edema and congestion, which contribute to the increased mortality rates [17]. 

Previous studies and a meta-analysis reported that MR is a strong predictor of mortality [18-21], and our results further support this (64.7% of STEMI patients with MR died during their hospital stay). However, the mortality rate in our study was much higher than that reported in three earlier studies (17.5% in Lahore, Pakistan and 29.4% in Birmingham, United Kingdom, 16.4% in Nepal) [22-24]. The higher mortality rate in the present study may be due to a smaller sample size and a study with a bigger sample size would give a closer rate to what has already been reported.

Limitations

Our study had a few important limitations. The sample size was relatively small, and it was conducted in only one hospital, both of which limit the generalizability of our results. Also, we were unable to screen participants for all relevant comorbidities, which may have introduced confounders.

## Conclusions

This study examined the impact of MR contributing to in-hospital mortality in STEMI patients. According to our results, patients with MR following MI have a poor prognosis. The relation between MR, complications, and mortality rates, informed decision making to adopt an appropriate modality of treatment requires a detailed assessment of the MR. Further studies are needed to evaluate the prognostic significance of treatment and preventive measures.
